# Sense of Time in Neurodevelopmental Disorders: ADHD and Developmental Dyscalculia from a Dimensional and Transdiagnostic Perspective

**DOI:** 10.3390/brainsci16020249

**Published:** 2026-02-23

**Authors:** Federica Cortesi, Sara Eralda Santirocchi, Rachele Montanelli, Lisa Toffoli, Andrea Gambarini, Gian Marco Marzocchi, Giovanna Mioni, Giovanni Mento, Anna Lucia Ogliari, Enrico Toffalini, Valentina Tobia

**Affiliations:** 1 Faculty of Psychology, Vita-Salute San Raffaele University, 20132 Milan, Italy; f.cortesi1@studenti.unisr.it (F.C.);; 2 Child in Mind Lab, Vita-Salute San Raffaele University, 20132 Milan, Italy; 3 NeuroDev Lab, Department of General Psychology, University of Padua, 35122 Padua, Italy; lisa.toffoli@unipd.it (L.T.); giovanni.mento@unipd.it (G.M.); 4 Department of Psychology, University of Milan-Bicocca, 20126 Milan, Italy; 5 Department of General Psychology, University of Padua, 35122 Padua, Italy; 6 Scientific Institute IRCCS E. Medea, 31015 Conegliano, Italy; 7 IRCCS San Raffaele Hospital, 20132 Milan, Italy

**Keywords:** sense of time, time processing, ADHD, developmental dyscalculia, transdiagnostic approach

## Abstract

**Highlights:**

**What are the main findings?**

- Children with a formal diagnosis of ADHD or DD showed lower SoT performance compared to typically developing peers.
- Attention and hyperactivity impairments had a greater association with SoT skills than mathematical difficulties alone.

**What are the implications of the main findings?**

- From a transdiagnostic perspective, SoT difficulties are better understood within dimensionally derived groups rather than categorical diagnostic groups.
- Assessing specific cognitive traits, such as SoT skills, could support a more tailored clinical approach, improving diagnostic accuracy, clinical utility, and intervention planning.

**Abstract:**

**Background:** Sense of Time (SoT) refers to a range of cognitive abilities involved in the time processing (TP) and management (TM) of temporal durations. Impairments in these abilities can impact various domains of daily life, including academic, social, and recreational activities. Recent evidence suggests that children with neurodevelopmental disorders (NDDs), particularly ADHD and Developmental Dyscalculia (DD), often exhibit deficits in time-related cognitive functions. Adopting a transdiagnostic approach, the present study investigates the relationships among SoT skills, inattention and hyperactivity, and mathematical difficulties, considering both categorical and dimensional perspectives. **Methods:** A total of 811 children completed SoT assessments (computerized tasks and self- and proxy-report questionnaires), mathematical tests, and questionnaires measuring attention and hyperactivity traits (self and proxy report). **Results:** Correlational analyses revealed significant associations between SoT and attention/hyperactivity, as well as between SoT and mathematical abilities. Group differences in TP and TM variables were examined using both categorical (diagnostic groups) and dimensionally derived group approaches. The results indicated more marked contrasts using the dimensional approach. Specifically, attentional and executive control difficulties appeared to have greater associations with TP and TM skills than mathematical difficulties alone. No evidence for attentional X mathematical difficulties interaction emerged. **Conclusions:** The SoT abilities were impaired in children with both ADHD and DD. A transdiagnostic dimensional approach allows for a more nuanced understanding of SoT profiles across different types of atypical development within the NDDs spectrum. Practical and clinical implications are discussed.

## 1. Introduction

Time is a cognitive construct that gives meaning to our perception of the world [1,2,3]. Every event and behavior unfolds within specific temporal intervals, and the ability to organize activities is crucial for adaptive functioning [4]. Given its pervasive role in daily life, Sense of Time (SoT) has been examined and can be defined as the set of cognitive abilities involved in time processing (TP) and time management (TM) of temporal durations. According to Grondin [1], these include the capacities to estimate, reproduce and discriminate time intervals, which represent fundamental skills that support more complex abilities such as time orientation and TM. Furthermore, the ability to keep track of the order and sequence of events is fundamental for both time orientation and TM and can be considered part of the broader construct of time cognition [5]. In the present study, the focus will be on cognitive processes that involve a component of explicit timing, specifically the accurate and conscious estimation, reproduction and discrimination of temporal intervals [5,6].

Primitive SoT is present early in life, yet children’s temporal judgments improve progressively throughout development [7,8,9], leading to greater accuracy in time estimation [2] and enhanced temporal discrimination ability [10]. Such improvement is primarily attributed to brain maturation [11,12], which supports cognitive abilities development [13,14,15,16,17]. Among these, selective attention and working memory play a particularly prominent role in temporal abilities. Both are entailed in the domain of executive functions (EF), a set of cognitive features that allow individuals to regulate their behavior to reach their purposes in their daily lives [18]. Selective attention supports both the initial reception of temporal stimuli and the monitoring of temporal information [11,19,20,21,22,23,24,25,26,27]. Working memory, in turn, plays a central role in time management [1,2,11,22,23,28,29] and underlies duration estimation [1,30,31]. Such executive component further contributes by holding temporal information online and sustaining the perception of temporal continuity [11,32,33].

Given that the accurate processing of temporal information is essential for human development and adaptive functioning [2,4,5], when these mechanisms are inefficient or compromised, individuals may encounter difficulties across multiple domains including timekeeping, activity planning [34], waiting behavior, and delayed responses or gratification [35]. Increasing evidence suggests that atypical TP may be observed in neurodevelopmental disorders (NDDs) [36,37]. NDDs are multifaceted conditions [38] defined as a heterogeneous group of disorders characterized by atypical development of cognitive, behavioral, and motor functions, typically emerging in childhood [39]. Despite their clinical diversity, these disorders often share impairments in cognitive processes [37], which have led researchers to adopt a transdiagnostic perspective, focusing on the underlying mechanisms that may cut across diagnostic categories rather than being unique to a single disorder [38,40]. In particular, the present work is underpinned by the Research Domain of Criteria (RDoC) framework [41]. This initiative aims to break down the specific sub-components of human functioning on several dimensional levels: lifespan, levels of expression and operationalizations.

Within this framework, a major source of temporal alteration in NDDs could arise from deficits in executive functions, as prominently observed in Attention Deficit/Hyperactivity Disorder (ADHD). ADHD is considered a lifelong condition in which symptom expression changes across developmental stages. Nevertheless, the disorder is consistently characterized by difficulties in one or more of three core domains—inattention, hyperactivity and impulsivity—that are disproportionate to an individual’s developmental level and interfere with daily functioning [39].

Beyond behavioral manifestations, individuals with ADHD frequently show impairments in executive components, including deficits in response inhibition, working memory and attentional control [42,43,44,45,46]. Neuroimaging studies further support these findings, revealing structural and functional anomalies within fronto-striato-parietal and fronto-cerebellar networks [46,47,48]. Alterations in these circuits are particularly relevant to temporal cognition, as they may underlie difficulties in monitoring, reproducing, and estimating time intervals [42,45,49,50]. Deficits in the executive domain may therefore disrupt the ability to generate consistent temporal estimates, contributing to the heightened variability and inaccuracy frequently observed in ADHD. In line with this view, Barkley [43] proposed that temporal deficits in ADHD may represent a secondary consequence of executive dysfunction, reflecting impaired self-regulation and cognitive control. In contrast, Sonuga-Barke [51] proposed a dual pathway model in which ADHD symptom heterogeneity and timing difficulties result from two independent patterns of deficits. The first pattern affects the executive domain, while the other, known as the Delay Aversion Theory (DAT) [52,53], is linked to the altered signaling of delayed rewards and delay aversion [51].

According to DAT [52], the tendency to perceive time in a biased way reflects a motivational tendency to avoid the aversive emotional state associated with waiting. Therefore, impulsive or premature responses in timing tasks may not only arise from executive dysfunction—as predicted by Schachar and colleagues [54]—but also from an underlying motivational bias toward immediate reward. This raises a central question about whether timing difficulties in ADHD are primarily the consequence of executive dysfunctions—such as impaired inhibitory control and working memory [25,35,42,45,49,50]—or whether they stem from a fundamental alteration in subjective time perception, where time is experienced as passing more slowly, leading to impatience and impulsive responses [15,45,55].

Over a decade ago, Sonuga-Barke and colleagues [56] revised the dual pathway model and proposed temporal cognition as a third dissociable neuropsychological pathway. Indeed, an altered SoT could underlie several problems of impulsiveness such as waiting behavior or delaying responses and gratification [57]. Therefore, temporal abilities were studied in preschoolers and were discovered as an early neuropsychological component of ADHD [58], suggesting that temporal cognition may represent an early-emerging cognitive vulnerability within the disorder.

However, time perception was also significantly associated with early academic performance and predicted reading and mathematical performance in preschoolers [58], highlighting that temporal difficulties may not be unique to ADHD among NDDs. Indeed, similar impairments have been observed in Specific Learning Disorders (SLD), defined as neurodevelopmental conditions characterized by persistent difficulties in the acquisition and use of academic skills—typically involving reading, writing, or mathematics—that fall substantially below age and educational expectations and cannot be explained by intellectual disabilities, uncorrected sensory deficits, inadequate instruction, or other neurological or psychiatric conditions [39].

Moll and colleagues [37] investigated SoT in SLDs and identified temporal anomalies in both Reading Disorder (RD) and Mathematics Disorder (MD). Specifically, timing deficits in RD appeared to be mediated by attentional difficulties, which may in turn contribute to executive function processes involved in temporal cognition. Importantly, these findings align with evidence showing that SLDs, despite presenting generally typical intellectual functioning, commonly show weaknesses in specific cognitive domains, most notably in terms of processing speed and working memory [59]. Since working memory is essential for maintaining temporal information and for discriminating among different time intervals [11,33], such domain-general weaknesses provide a plausible explanation for the temporal difficulties observed across SLDs subtypes. However, conflicting evidence suggests that difficulties in SoT cannot be attributed exclusively to lapses of attention, reduced alertness, or generally noisy processing [60]. In RD, temporal difficulties may also reflect domain-specific mechanisms related to language processing. The core deficit in RD is phonological, and therefore the mechanisms underlying the explicit encoding and decoding of phonological information must be considered to properly understand the temporal structure of language [60].

Moreover, children with MD seem to show even more pronounced impairments in temporal cognition than their peers with RD [37], suggesting that Developmental Dyscalculia (DD) may be particularly associated with deficits in SoT abilities. In the DSM-5-TR [39], DD is described as a selective impairment in the development of arithmetic skills, involving difficulties in basic calculation, understanding numbers, grasping numerical concepts and relationships, and processing arithmetic operations.

In relation to SoT, individuals with DD often report subjective difficulties in perceiving temporal information, which manifest as challenges in time management [34]. This raises the question of whether such temporal difficulties stem from anomalies in number cognition or rather reflect intrinsic alterations in time perception and temporal cognition [34]. One of the most influential accounts addressing this issue is the A Theory of Magnitude (ATOM) [61], which proposes that time, number and space rely on partially shared magnitude representation systems. According to this framework, numeracy emerges from learned associations between different quantitative dimensions, such as time and number, both rooted in a common magnitude system present from birth. Neuroimaging studies provide support for this hypothesis, demonstrating that the intra-parietal sulcus (IPS) is involved in both time representation and arithmetic processing, suggesting the existence of a shared neural substrate [62].

Overall, SoT emerges as an overlapping domain of vulnerability in DD and ADHD. Indeed, research suggests that both NDDs are characterized by SoT alterations across multiple dimensions—from temporal reproduction and discrimination to estimation and management—although the underlying mechanisms may differ. While ADHD timing difficulties are often attributed to deficits in inhibitory control and executive regulation [13,35,54], those observed in DD seem more closely linked to magnitude processing [63,64].

However, studies directly addressing the shared temporal mechanisms between the two NDDs remain scarce. Within this framework, SoT has been proposed as a potential cognitive dimension that could link common neurocognitive pathways across multiple NDDs, while also allowing for the characterization of disorder-specific patterns in the nature, degree, and development of SoT alterations [7,37].

The present study aims to expand the current knowledge of SoT and NDDs by adopting both categorical and dimensional approaches. From a categorical approach, SoT will be systematically investigated in participants with a clinical diagnosis of DD and/or ADHD, to identify both overlapping and disorder-specific patterns of temporal processing. From a dimensionally defined groups approach, SoT performance will be examined as a continuous trait on the entire sample, selecting those with a deficit in mathematical performance and inattention/hyperactivity, regardless of formal diagnosis. In addition, analyses will control for general cognitive ability to ensure that the observed differences in SoT are not attributable to broader intellectual functioning. We expected SoT impairments to be associated with both mathematical and attentional weaknesses, with partially distinct patterns reflecting the specific cognitive profiles involved. It is hypothesized that adopting a dimensional approach may “tighten the mesh of the net”, thereby enabling a more fine-grained focus on the specific cognitive processes and abilities involved in SoT.

## 2. Methods

### 2.1. Participants

Participants were recruited from schools and clinical centers in Milan and Padua that joined the research project. From May 2024 to November 2025, students in primary and middle schools were assessed during school hours. During the summer period of 2025, individual assessments to children with NDDs were carried out at clinical centers in Milan and Padua.

A total of 811 children (6–13 years old, mean age = 9.2 ± 1.59 years old, and 49.2% female) have been included in the present study. Proxy-report assessments were available for 84.63% of children for teacher reports and 59.23% for parent reports. Parents reported the presence of a certified diagnosis of ADHD and/or SLDs by completing the appropriate questionnaire. While we did not have direct access to the clinical reports, assessments of ADHD and SLD in Italy are carried out by dedicated multidisciplinary specialists who operate in accordance with nationally standardized procedures [65] and internationally recognized diagnostic criteria (ICD–DSM), which ensure a relatively consistent and structured diagnostic process. A total of 48 children with ADHD (6–13 years old, mean age = 9.7 ± 1.53 years old, and 19.6% female) and 24 children with DD (8–13 years old, mean age = 11.61 ± 1.28 years old, and 58.3% female) were recruited principally through clinical centers, but also through schools. Three of these children have comorbid ADHD and DD. Given the high comorbidity and co-occurrence of NDDs [66], the presence of another SLD (e.g., dyslexia) was not considered an exclusion criterion. Among participants with a formal diagnosis, 13.33% had an additional diagnosis of developmental dyslexia.

### 2.2. Instruments and Procedures

At the schools, participants were assessed in both collective and individual settings. Some tests were administered in classrooms, as they did not require individual interaction with the evaluators (e.g., the KBIT-2 test and self-report questionnaires). The order of tests in the collective setting was the same for all the participants. Individual evaluations at the schools were conducted in separate rooms, with one evaluator per participant. At the clinical centers, all tests were administered individually. The parents and teachers of the respective participants completed online proxy-report questionnaires, each lasting approximately 10 min. Before taking part in the study, the children’s parents and teachers signed an informed consent form and a data processing agreement. The research was conducted in agreement with the guidelines of the Declaration of Helsinki and was approved by the Institutional Review Board of psychological research (Area 17) at the University of Padua (protocol code 312-a; 19 January 2024).

### 2.3. Ad Hoc Computerized Tasks

Based on a recent systematic review [67], three computerized tasks were selected and developed to cover the measurement range of time processing skills as broadly as possible: Time Estimation (TE), Time Reproduction (TR) and Time Discrimination (TD) tasks.

The TE task [68] assesses the ability to estimate the duration of two 30 s videos, created using AI. The videos, which differ visually (thief and policeman; boat sailing) and auditorily (respectively, fast-paced vs. slow background music), are presented via PC. No instructions were provided regarding strategies to avoid (e.g., counting), in order not to constrain the spontaneous adoption of strategies by participants. After each video is played, the participant must write the number of seconds corresponding to the duration of each video in a form. Performance was calculated as the mean absolute deviation from the target.

The TR task assesses the ability to reproduce visually presented time durations. Developed using PsychoPy (version 2023.2.3) [69], the TR task consists of a light bulb that turns on for 11 target time durations (2–12 s, in 1 s steps), presented in random order. When the light bulb turns off, the participant must press the spacebar to turn it on again for the same amount of time as the target stimulus [63]. Performance was calculated as the mean absolute deviation from the target.

The TD task assesses the ability to discriminate between two auditory stimuli (lasting between 0.3 and 9 s) presented on a PC, each paired with one visual stimulus. Developed using PsychoPy (version 2023.2.3) [69], the TD task is composed by 36 trials, each of which consists of the presentation of two auditory stimuli (with a 400 Hz sine wave) coupled with an image of an animal wearing headphones, “as if it was listening to music with the participant”. At the end of the trial, the images of the animals are shown side by side and the participant must indicate which animal “heard” the longer sound by pressing one of two keys on the keyboard [63]. The ratio of stimuli durations adapts according to the participant’s performance. The task gradually becomes more difficult (lower ratio) and in the event of a reversal (i.e., incorrect response), the next trial moves to a higher ratio. These points are referred to as reversals. Performance is calculated by discarding the first two reversals and averaging the last six reversals or all available reversals if fewer than six occur.

**Figure 1:**
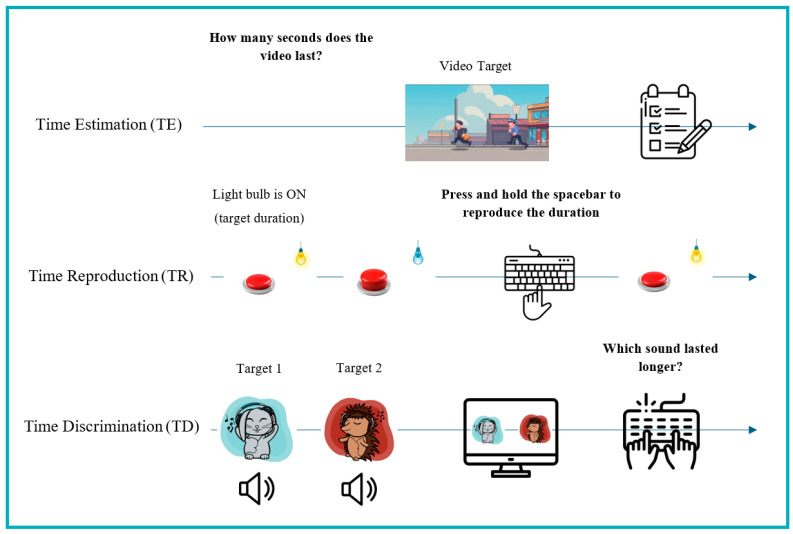
Ad -hoc computerized tasks to assess time-processing skills.

Since all task scores originally return a deviation score (a higher score corresponds to a worse performance), the scores were standardized to align with the other measures used in this study by applying a sign reversal. Figure 1 illustrates the visual stimuli and summarizes the computerized time processing tasks.

### 2.4. Self- and Proxy-Report Questionnaires

The Time Management and Orienting Questionnaire (TMOQ) [70,71] assesses children’s time orientation and time management skills. It includes three versions: a self-report questionnaire for children and two proxy-report versions for parents and teachers. The TMOQ_child consists of 16 both open- and closed-ended questions organized into two subscales: (1) time orientation (e.g., “How many minutes are there in an hour?”) and (2) time management (e.g., “Do you think you can do three pages of maths exercises and have dinner in ten minutes?”). The TMOQ_parent and TMOQ_teacher each include ten items (e.g., “If given a time limit for an activity, can they complete it before the time is up?”) rated on a four-point Likert scale ranging from zero (never) to three (very often). Overall, the TMOQ proxy reports return an assessment of the child’s general semantic knowledge about time as well as their understanding of their own time-related abilities. For each informant, a total score is calculated as the sum of the item responses.

Self- and proxy-report questionnaires from the Batteria italiana per l’ADHD—Revised (BIA-R) [72] were also administered to assess inattention- and hyperactivity-related behaviors. These included the self-report Scala per i Disturbi di Attenzione/Iperattività per Bambini (SDAB; child version), the parent version (SDAG), and the teacher version (SDAI). The SDAB consists of 14 items, whereas the SDAG and SDAI each include 18 items. Responses are provided on a four-point Likert scale ranging from ‘never’ to ‘very often’. All three questionnaires produce two scores, based on the sum of the item responses: one for inattention and one for hyperactivity/impulsivity.

The time processing tasks and the three versions of the TMOQ (in Italian) are freely accessible on the Time Mastery Project website (https://www.timemasteryproject.com/).

### 2.5. Neuropsychological Test

As an index of non-verbal reasoning, a selection of 40 matrices from the KBIT-2 [73] was administered to the participants. Within a 10 min time limit, participants were required to complete as many matrices as possible. The total number of correct responses was used to calculate the score of nonverbal reasoning.

### 2.6. Mathematical Abilities

The Numerical Reasoning test (AC-MT-3) [74] assesses the ability to understand relationships between numbers and logical–mathematical reasoning. Participants complete numerical sequences by selecting the correct number from multiple options, based on the arithmetic pattern of the sequence. Performance is measured as the number of correct responses provided within a 2 min time limit.

The Counting test (BDE-2) [75] assesses the ability to count quickly and accurately, first in ascending order and then in descending order. The execution time is recorded, and the number of correctly counted digits is measured.

The Mental Calculation test (BDE-2) [75] assesses the ability to perform mathematical operations without external help. A total of 18 operations, including additions and subtractions of increasing difficulty depending on grade level, are administered. The number of correct responses is calculated.

### 2.7. Statistical Analysis

#### 2.7.1. Data Standardization for Norming and Dimensional Analyses Purposes

Prior to statistical analysis, raw SoT scores were standardized using the school-based community sample, excluding children enrolled within the clinical centers. Participants with extreme scores were identified using scatterplots and the skewness values of the SoT variables. Based on this inspection, 17 participants with raw scores exceeding 80 s in the TE task or 175 s in the TR task were considered outliers and excluded from the analyses. After their removal, the skewness of all SoT variables was below two, indicating an acceptable distribution. Standardized scores (z-scores) were calculated separately for each grade using grade-specific means and standard deviations for TP tasks, TMOQ scores and for the KBIT-2 variable. Scores assessing inattention and hyperactivity, as well as raw mathematics scores, were standardized according to the normative data provided by the respective manuals (AC-MT-3 [74]; BIA-R [72]; and BDE-2 [75]). In cases of a lack of teacher report norms for the seventh and eighth grades, norms for the sixth grade were used. The z-scores were used in all subsequent analyses.

Pearson correlations with Bonferroni corrections were run between SoT variables, and the measures of inattention and hyperactivity reported by the participants, parents, and teachers, as well as between the SoT variables and measures of mathematical abilities. To ensure consistent directionality across the measures, standardized inattention and hyperactivity scores were sign reversed (multiplied by −1), so that lower z-scores always reflected higher symptom severity (worse functioning), and impairment could be defined as z < −1.5.

The standardized scores from the six inattention and hyperactivity measures were classified into two levels: impaired (<−1.5 SD) and typical (>−1 SD). The participants originally recruited both from the general population and from the clinical sample, all showing impairments in at least 66.7% (over half) of the measures, were classified into the Attention/Hyperactivity Impairment group (AH/IMP; *n* = 47). The same procedure was applied to the three mathematics measures, and participants showing impairments in at least 66.7% (over half) of the measures were classified into the Mathematical Impairment group (MATH/IMP; *n* = 32). In the same fashion, six participants were classified into the AH/IMP + MATH/IMP group. A control group (CG1; *n* = 70), matched for gender and grade, was randomly selected. The participants in this control group showed typical performance (>−1 SD) in at least seven out of nine measures of inattention, hyperactivity, and mathematics. This approach allowed the CG1 to retain some within-group variability rather than being treated as fully homogeneous.

#### 2.7.2. ANCOVAs

##### Dimensional Approach

A series of 2 × 2 analyses of covariance (ANCOVAs) was conducted with SoT measures as the dependent variables, and two between-participants factors: Attention/Hyperactivity Impairment (AH/IMP vs. non-AH/IMP) and Mathematical Impairment (MATH/IMP vs. non-MATH/IMP); non-verbal reasoning (KBIT-2 z-score) was the covariate.

##### Categorical Approach

A second control group (CG2; *n* = 70), matched for gender and grade, was selected for comparisons with the diagnostic groups. As well as CG1, participants in this group showed typical performance (>−1 SD) in at least seven out of nine measures of inattention, hyperactivity, and mathematics. This approach allowed CG2 to retain some within-group variability rather than being treated as fully homogeneous.

Additional 2 × 2 ANCOVAs were conducted with SoT measures as dependent variables, and two between-participants factors: ADHD diagnosis (ADHD vs. non-ADHD) and DD diagnosis (DD vs. non-DD); again, KBIT-2 z-score was the covariate. Pairwise contrasts comparisons were performed where appropriate.

## 3. Results

Table 1 reports the descriptive statistics.

The results of the correlations between the standardized SoT variables and the measures of inattention and hyperactivity, and the SoT variables and mathematical ability measures are shown in Table 2 and Table 3, respectively. The Bonferroni corrections were set at 0.0013 (for variables in Table 2) and 0.0027 (for variables in Table 3). The inattention and hyperactivity variables, as well as the mathematical variables, correlate with different weights and levels of significance depending on the SoT variable considered. This underlines the heterogeneity and complexity of the SoT construct.

### 3.1. Sense of Time Skills in Dimensionally Defined Groups

Table 4 shows the performance of the four groups (CG1, AH/IMP, MATH/IMP and AH/IMP + MATH/IMP) in terms of the mean and the standard deviation with respect to the SoT variables. Table 4 also shows the group effects and contrasts. The non-verbal reasoning covariate (KBIT-2 z-score) was significant in all analyses (F ranging from 7.423 to 22.026; *p* from 0.007 to < 0.001), except for the TE- and TMOQ_p-dependent variables. MATH/IMP and AH/IMP effects were significant for some SoT variables, while their interaction was not significant. Contrasts indicated that the AH/IMP + MATH/IMP group performed worse than the MATH/IMP group (*p* < 0.05) for TD and TMOQ_t. The CG1 group performed better than the three clinical groups (*p* < 0.05).

### 3.2. Sense of Time Skills in Diagnostic Groups

**Figure 2:**
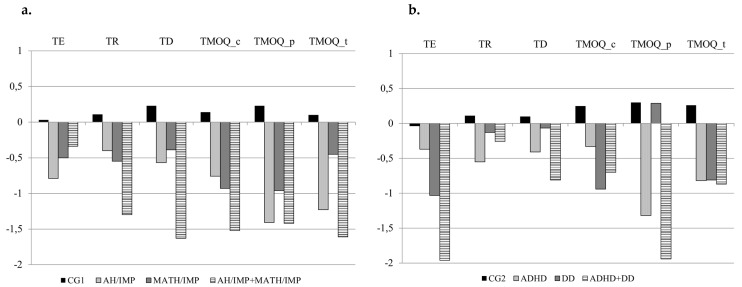
Standard Mean Scores of the Four Groups on Sense of Time Variables. Note. (**a**) Dimensionally based group. (**b**) Diagnosis-based group.

Table 5 shows the performance of the four groups (CG2, ADHD, DD and ADHD + DD) in terms of the mean and the standard deviation with respect to the SoT variables. Table 5 also shows the group effects and contrasts. The non-verbal reasoning covariate (KBIT-2 z-score) was significant in all analyses (F ranging from 4.534 to 16.977; *p* from 0.036 to <0.001) except for the TE- and TMOQ_p- dependent variables. ADHD and DD effects were significant for some SoT variables, while their interaction was not significant. Contrasts indicated that the ADHD + DD group performed worse than the DD group (*p* < 0.01) on the TMOQ_p variable. The CG2 group performed better than the three clinical groups (*p* < 0.05). The analyses were also conducted using a group of participants with ADHD without comorbidity with other SLDs (i.e., dyslexia, spelling disorder, and dysgraphia). The ANCOVAs showed no significant differences. Therefore, it was decided to include the ADHD group with comorbid SLDs to obtain a larger sample size. Figure 2 summarizes the groups’ z-scores on all time-related variables, showing (a) the dimensionally defined groups and (b) the diagnosis-based groups.

## 4. Discussion

Building on evidence suggesting that Sense of Time represents an overlapping domain of vulnerability across NDDs, the present study examined temporal cognition in DD and ADHD using both categorical and dimensional approaches. By comparing diagnostic groups and, in parallel, investigating mathematical difficulties (MATH/IMP) and inattention/hyperactivity (AH/IMP) as continuous dimensions, this study aimed to clarify whether SoT alterations reflect disorder-specific mechanisms or shared neurocognitive processes that cut across diagnostic boundaries.

### 4.1. Associations of SoT Skills with Inattention, Hyperactivity and Mathematical Abilities

Considering inattention and hyperactivity variables, the correlational analyses indicate that timing difficulties are component specific rather than global, highlighting a differentiated pattern across SoT skills. The absence of significant associations between TE and inattention or hyperactivity suggests that this component of time processing may be less sensitive to attentional–executive variability than other TP tasks. Given that TE is thought to rely more strongly on perceptual encoding mechanisms, this pattern is consistent with the view that ADHD-related traits are more strongly expressed in those TP components that place higher demands on attention and working memory, rather than in duration encoding per se [76]. A different pattern emerged for the TR task, which requires sustained attentional engagement and the continuous maintenance of temporal information. The presence of an association between TR, and inattention and hyperactivity suggests a role for executive (dys)function—particularly selective attention and working memory—in shaping TP alterations [76,77,78]. Furthermore, the increased variability in the reproduced intervals may reflect difficulties in maintaining stable temporal representations over time rather than an impaired time perception per se. Similarly, the performance of the TD task was significantly related to both inattention and hyperactivity. Indeed, this task requires sustained attention and the comparison of successive temporal intervals, placing demands on executive control [77]. This result supports the interpretation that attentional fluctuations and reduced cognitive control contribute to TP impairments. Interestingly, significant correlations emerged only for the teacher version (SDAI) of the BIA-R questionnaire. According to previous findings by Tobia et al. [71] and Tombini et al. [12], teacher reports may provide a more reliable index of children’s temporal abilities, as teachers are able to compare a child’s performance with that of same-age peers and to observe SoT-related behaviors across a range of time-constrained activities [71]. For TM, correlational analyses revealed a robust association between both inattention and hyperactivity traits as reported by children, parents and teachers. Because time management relies on the ability to plan, monitor, and regulate goal-directed behavior over time, these findings are consistent with evidence highlighting TM difficulties in academic settings [79], and impairments in waiting behaviors and delayed response gratification [35].

Considering mathematical abilities, the correlational analyses revealed a robust association between TP and mathematical performance, in line with ATOM [61]. Both Numerical Reasoning and Mental Calculation were associated with performance across all TP tasks, suggesting that deficits in general magnitude processing—affecting both symbolic and non-symbolic representations—may interfere also with temporal cognition in individuals with math impairments or DD [61,64]. In contrast, counting abilities were selectively associated with TR and TD, but not with TE. This pattern suggests that counting may serve as a supportive strategy in tasks that allow for the segmentation and comparison of temporal intervals [80,81,82]. However, this result is not straightforward, given that the TE task involves longer durations (30 s), which would in principle allow for greater opportunity to rely on counting strategies. One possible explanation is that the BDE counting task, which is based on speeded-forward and -backward counting, may index not only counting skills per se but also processing speed and temporal updating. These components are particularly relevant for TR and TD, which require the rapid encoding, comparison, and updating of successive short intervals, but may play a less central role in supra-second estimation, which relies more strongly on perceptual and memory-based mechanisms [76]. Moreover, the absence of a significant association between counting abilities and TE performance suggests that, even when spontaneously adopted, counting strategies may play a limited role in supra-second estimation in children. Significant correlations were also observed between mathematical abilities and TM. Notably, this association emerged for the child version of the questionnaire, in line with the subjective dimension of time management impairments in individuals with DD reported by Cappelletti and colleagues [34]. Moreover, the absence of a relationship with parent reports further supports the notion that teachers may be more optimal raters than parents for SoT skills [12,71].

The results of the correlational analyses showed that SoT is associated both with levels of inattention and hyperactivity (self and proxy reported), and with mathematical abilities. These findings suggest that SoT impairments are not a trait specific to a single diagnostic category but rather may reflect continuous variations in cognitive domains that can be partially overlapping.

### 4.2. Sense of Time: Comparing Dimensionally Derived and Categorical Groups

The present study adopts a transdiagnostic perspective in relation to SoT, combining both categorical and dimensional approaches to better understand the variations in this construct across groups defined either by formal diagnoses or by continuous differences in mathematical and/or attentional profiles. Although the dimensional approach involved the formation of groups, these groups were defined based on continuous variation in attentional/hyperactivity and mathematical functioning rather than on diagnostic status. As such, they represent groupings on underlying dimensions rather than diagnostic categories, and therefore constitute dimensional operationalizations of neurodevelopmental traits rather than categorical nosological entities, in line with contemporary transdiagnostic frameworks such as HiTOP [83] and recent proposals to conceptualize neurodevelopmental conditions within a dimensional spectrum [84].

When comparing the performance of the four dimensionally derived groups (CG1; AH/IMP; MATH/IMP; and AH/IMP + MATH/IMP), distinct performance profiles emerged for the SoT skills associated with specific combinations of inattention and hyperactivity and/or mathematical abilities. Overall, the control group outperformed all other groups across the tasks, supporting the view of SoT as a domain of vulnerability across NDDs traits. More specifically, TR as well as all TM skills were globally impaired both in the AH/IMP group and MATH/IMP group compared to CG1, suggesting that SoT relies on both executive control processes [77,79] and non-symbolic magnitude comparison mechanisms [64]. Interestingly, the AH/IMP + MATH/IMP group showed more severe impairments than the MATH/IMP group in TD and in the TMOQ_teacher. This contrast indicates that the co-occurrence of attentional and hyperactivity-related difficulties is associated with a greater level of impairment in these SoT skills than mathematical difficulties alone, while mathematical difficulties remain related to a weaker performance in both TP and TM. Effect sizes ranged from moderate to large, with particularly pronounced effects for the TMOQ_parent variable.

When comparing performance of the four diagnostic groups (CG2; ADHD; DD; and ADHD + DD), the resulting patterns are less clearly delineated than those emerging from the dimensionally based approach. More specifically, CG2 outperformed all clinical groups in less SoT tasks (only in TE, TD, and in TM parent version—TMOQ_parent). Compared to CG2, the DD group showed impairments only in TE, reflecting the influence of numerical and counting deficits within this specific domain [34], which typically characterize individuals with this SLD. In contrast, the ADHD group exhibited persistent difficulties in TD and in TMOQ_p, further supporting the central role of executive control processes in this TP domain [77] and in TM. Furthermore, the comorbidity group (DD + ADHD) showed more severe impairments than the DD group only in TMOQ_parent. This contrast highlights the strongest influence of a diagnosis of ADHD on TM compared to DD. Categorical diagnostic comparisons highlighted more general differences between groups, showing reduced sensitivity in capturing inter-group variability and overlaps among cognitive profiles. As for the dimensionally derived groups, effect sizes ranged from moderate to large, with particularly pronounced effects for the TMOQ_parent variable. Moreover, the internal heterogeneity of the ADHD and DD diagnostic groups may have contributed to the performance overlaps with the control groups and to a reduced number of main effects, which instead emerged in the dimensionally derived group comparisons.

Across both categorical and dimensional approaches, the effect of ADHD on TMOQ_parent was a robust and consistent finding. These results suggest that, beyond diagnostic or process-related factors, behavioral aspects observable in everyday and home contexts may be closely linked to SoT performance.

### 4.3. Sense of Time in a Transdiagnostic Perspective

The present study aims to consider SoT as a transdiagnostic dimension within NDDs. The presence of difficulties related to rhythm and timing in NDDs has already been hypothesized and reviewed by Lense et al. [85], who focused on specific disorders, including ADHD and developmental dyslexia. Previous studies have also highlighted the existence of temporal impairments in school-age children with NDDs such as ADHD [42] and SLDs affecting mathematical abilities [86]. Most of these studies compared the profiles of children with a diagnosis with those of their typically developing peers.

To the best of our knowledge, the present study represents the first attempt to examine the SoT construct by simultaneously considering specific traits of ADHD and DD. This approach is expected to situate the study within a broader framework characterized by growing interest in dimensional models of psychopathology. These include models such as RDoC [87] and HiTOP [83], which reconceptualize disorders not as discrete categories but as dimensions along a continuum. Within this framework, we tested the hypothesis advanced by Poletti et al. [88], according to which NDDs such as ADHD and DD can be conceptualized as partially overlapping manifestations within a broader neurodevelopmental spectrum [84].

Inattention and hyperactivity, as well as learning disorders—in the present study operationalized as difficulties in mathematical abilities—have been proposed as some of the possible traits and dimensions defining the phenotypes of neurodevelopmental disorders [88]. Accordingly, the results of the present study allow us to move beyond simple associations between diagnostic labels and SoT deficits, and instead to examine how the presence of specific phenotypic traits, typical of NDDs, may be linked to distinct profiles of SoT impairment. Consistent with the study by Sonuga-Barke et al. [56], 44.2% of the sample with an ADHD diagnosis showed difficulties in time processing. This result highlights the informative value of diagnostic labels for identifying disorder-related vulnerabilities but also underscores their limitations in capturing the substantial heterogeneity present within diagnostic groups.

### 4.4. Implications for Practice and Future Perspectives

The use of a dimensional approach is interesting not only for considerations strictly related to research, but also within a clinical psychodiagnostics context. Moving toward an increasingly tailored approach, the assessment of cognitive components—including, for example, SoT skills—can lead to a more detailed understanding of NDDs. Although the simplicity of the categorical approach allows mental health clinicians to communicate in a clear and accessible manner [89], adopting a dimensionally derived groups perspective makes it possible to improve not only the diagnostic accuracy, but also clinical utility and intervention planning [88].

As suggested by a recent systematic review [67], identifying strengths and weaknesses in SoT abilities in children with NDDs can guide and inform the selection of specific compensatory tools. These tools, such as visual timers or structured time management aids [90], can be used by clinicians and schools to support autonomy and self-efficacy. In the future, it would be interesting to conduct longitudinal studies to broaden the perspective not only “horizontally”—that is, by adopting a dimensional approach—but also “vertically,” to examine potential changes in SoT abilities across development and growth in individuals with NDD profiles. Furthermore, it would be interesting to include a wider range of traits to define the phenotypes of NDDs, allowing for a more detailed profiling of the neurodevelopmental spectrum. Finally, future studies could investigate whether executive functions mediate or moderate the relationship between attentional/hyperactivity traits and SoT outcomes.

### 4.5. Limitations

Some limitations of the current study need to be considered. First, the cross-sectional and correlational design does not allow for inferences about the directionality or causal mechanisms linking SoT, attentional traits, and mathematical abilities. Although the observed patterns are consistent with theoretical models emphasizing executive and magnitude-related processes, longitudinal and experimental studies will be necessary to clarify how these components interact over development. Second, diagnostic status was based on parent reports rather than on direct access to clinical records, and the clinical groups, particularly the DD and ADHD + DD groups, were relatively small, due to resource constraints [91]. This may have reduced statistical power and increased within-group heterogeneity, which could partly account for the reduced sensitivity of categorical comparisons relative to the dimensional approach. Third, reading and writing skills were not directly assessed in the present study, which limited our ability to further explore the relationship between developmental dyslexia and SoT. Although the study focused on dyscalculia, future research could more specifically examine other SLDs and determine whether impairments in reading and writing skills may affect SoT abilities. Fourth, the findings for TM relied on self- and proxy-report measures, which may be influenced by contextual and subjective factors and therefore do not reflect pure cognitive performance; however, these measures also provide important ecologically valid information about how children manage time in everyday life, which is a central aspect of SoT functioning beyond laboratory-based tasks. Fifth, the choice of a dimensionally derived group analysis balances theoretical dimensionality with methodological interpretability, allowing us to test dimensional hypotheses while remaining comparable with previous group-based studies [67]. Future research could build on this approach by using fully continuous statistical methods. Sixth, the small number of participants in the comorbid AH/IMP + MATH/IMP group (n = 6) may have limited the statistical power of the contrasts, increasing the risk of Type II errors; however, effect sizes were quantified using Cohen’s *d* to support the interpretation of the results. Finally, the present study focused primarily on explicit timing. In the future, it will be interesting to explore implicit timing abilities in NDDs.

## 5. Conclusions

The present study adopts a transdiagnostic perspective on SoT skills by combining both categorical and dimensional approaches. Overall, the findings suggest that categorical and dimensional frameworks are not mutually exclusive; rather, a dimensionally derived groups approach provides a more nuanced and fine-grained understanding of the relationship between SoT and complex cognitive domains. In particular, the co-occurrence of high levels of inattention and hyperactivity together with mathematical difficulties is associated with an additive effect, resulting in greater impairment in SoT skills. Interestingly, attentional and executive control difficulties have a stronger association with TP and TM skills than mathematical difficulties alone. Together, these findings support the view of SoT as a transdiagnostic domain of vulnerability, whose profiles are better captured by dimensional traits compared to by diagnostic labels alone, and highlight the potential value of SoT as a clinically meaningful marker across the neurodevelopmental spectrum.

## Figures and Tables

**Table 1:** Descriptive statistics.

	Min	Max	M	DS	Skewness (SE)	Kurtosis (SE)
Age	5.69	13.4	9.20	1.59	0.39 (0.086)	0.01 (0.171)
KBIT-2	3.00	40.00	26.80	6.70	−0.691 (0.086)	0.477 (0.172)
TE	0.00	70.00	11.35	9.65	1.88 (0.086)	6.26 (0.17)
TR	3.62	172.83	31.61	25.74	1.83 (0.087)	3.98 (0.17)
TD	1.00	3.00	1.55	0.49	1.28 (0.088)	0.71 (0.17)
TMOQ_c	2.00	16.00	11.41	2.61	−0.59 (0.099)	0.08 (0.19)
TMOQ_p	0.00	30.00	22.39	6.27	−0.94 (0.093)	0.45 (0.18)
TMOQ_t	5.00	30.00	22.72	5.35	−0.71 (0.111)	−0.19 (0.22)

Note. “TE”, Time Estimation task; “TR”, Time Reproduction task; “TD”, Time Discrimination task; “TMOQ_c”, Time Management and Orienting Questionnaire, child version; “TMOQ_p”, Time Management and Orienting Questionnaire, parent version; and “TMOQ_t”, Time Management and Orienting Questionnaire, teacher version.

**Table 2:** Pearson correlations between the SoT Tests and the inattention and hyperactivity variables.

N (329–679)	SDAB_IA	SDAB_HI	SDAI_IA	SDAI_HI	SDAG_IA	SDAG_HI
TE	−0.070 *p* = 0.088	−0.009 *p* = 0.823	−0.207 *p* = 0.157	0.115 ** p = 0.003	−0.144 ** p = 0.002	−0.104 * *p* = 0.023
TR	−0.021 *p* = 0.621	−0.033 *p* = 0.427	−0.196 ** ***p* < 0.001**	−0.139 ** ***p* < 0.001**	−0.104 * *p* = 0.026	−0.085 *p* = 0.069
TD	−0.091 * p = 0.030	−0.126 ** p = 0.003	−0.283 ** ***p* < 0.001**	−0.134 ** ***p* < 0.001**	−0.074 *p* = 0.115	−0.042 *p* = 0.368
TMOQ_c	−0.216 ** ***p* < 0.001**	−0.153 ** ***p* < 0.001**	−0.405 ** ***p* < 0.001**	−0.134 ** p = 0.003	−0.157 ** p = 0.004	−0.044 *p* = 0.425
TMOQ_p	−0.244 ** ***p* < 0.001**	−0.261 ** ***p* < 0.001**	−0.392 ** ***p* < 0.001**	−0.199 ** ***p* < 0.001**	−0.560 ** ***p* < 0.001**	−0.495 ** ***p* < 0.001**
TMOQ_t	−0.233 ** ***p* < 0.001**	−0.167 ** ***p* < 0.001**	−0.641 ** ***p* < 0.001**	−0.275 ** ***p* < 0.001**	−0.327 ** ***p* < 0.001**	−0.209 ** ***p* < 0.001**

Note. All scores are standardized (z-scores). “IA”, Inattentive subscale; “HI”, Hyperactive–Impulsive subscale; “TE”, Time Estimation task; “TR”, Time Reproduction task; “TD”, Time Discrimination task; “TMOQ_c”, Time Management and Orienting Questionnaire, child version; “TMOQ_p”, Time Management and Orienting Questionnaire, parent version; and “TMOQ_t”, Time Management and Orienting Questionnaire teacher version. **. The correlation is significant at the 0.01 level (two-tailed). *. The correlation is significant at the 0.05 level (two-tailed). Bold correlations are significant at the *p* < 0.0013 level following Bonferroni correction.

**Table 3:** Pearson correlations between the SoT Tests and the mathematical variables.

N (194–415)	Counting	Numerical Reasoning	Mental Calculation
TE	0.104 * p = 0.035	0.166 ** ***p* = 0.001**	0.201 ** ***p* < 0.001**
TR	0.178 ** ***p* < 0.001**	0.165 ** ***p* = 0.001**	0.238 ** ***p* < 0.001**
TD	0.165 ** ***p* < 0.001**	0.166 * ***p* = 0.001**	0.216 ** ***p* < 0.001**
TMOQ_c	0.224 ** ***p* < 0.001**	0.381 ** ***p* < 0.001**	0.414 ** ***p* < 0.001**
TMOQ_p	0.187 ** p = 0.005	0.125 *p* = 0.083	0.189 ** p = 0.004
TMOQ_t	0.218 ** ***p* < 0.001**	0.313 ** ***p* < 0.001**	0.302 ** ***p* < 0.001**

Note. All scores are standardized (z-scores). “TE”, Time Estimation task; “TR”, Time Reproduction task; “TD”, Time Discrimination task; “TMOQ_c”, Time Management and Orienting Questionnaire, child version; “TMOQ_p”, Time Management and Orienting Questionnaire, parent version; and “TMOQ_t”, Time Management and Orienting Questionnaire, teacher version. **. The correlation is significant at the 0.01 level (two-tailed). *. The correlation is significant at the 0.05 level (two-tailed). Bold correlations are significant at the *p* < 0.0027 level following Bonferroni correction.

**Table 4:** Means, Standard Deviations, and ANCOVA Results for SoT Tests by dimensionally derived groups (KBIT2-adjusted).

	CG1 (30% F)	AH/IMP (19.14% F)	MATH/IMP (46.87% F)	AH/IMP+ MATH/IMP (33.33% F)	AH/IMP Effect		MATH/IMP Effect		AH/IMP+ MATH/IMP effect		Contrasts
	M (SD)	M (SD)	M (SD)	M (SD)	F (dfs)	ɳ^2^p	F (dfs)	ɳ^2^p	F (dfs)	ɳ^2^p	CG1 vs. AH/IMP vs. MATH/IMP vs. AH/IMP+MATH/IMP
TE	0.03 (1.09)	−0.79 (2.35)	−0.50 (1.37)	−0.34 (1.43)	0.744 (1;149)	0.005	0.020 (1;149)	0.001	1.545 (1;149)	0.010	-
TR	0.11 (0.75)	−0.40 (1.26)	−0.55 (1.49)	−1.30 (1.39)	4.531 (1;145) *	0.030	5.257 (1;145) **	0.035	0.215 (1;145)	0.001	CG1 > AH/IMP (*d* = 0.52), MATH/IMP (*d* = 0.64)
TD	0.23 (0.79)	−0.57 (1.25)	−0.39 (1.16)	−1.63 (1.83)	14.457 (1;145) ***	0.089	5.840 (1;145) *	0.039	0.882 (1;145)	0.006	CG1 > AH/IMP (*d* = 0.80), MATH/IMP > AH/IMP+MATH/IMP (*d* = 0.97)
TMOQ_c	0.14 (1.02)	−0.76 (0.98)	−0.93 (1.34)	−1.52 (1.10)	5.148 (1;109) *	0.045	5.897 (1;109) *	0.051	0.212 (1;109)	0.002	CG1 > AH/IMP (*d* = 0.89), MATH/IMP (*d* = 0.93)
TMOQ_p	0.23 (1.07)	−1.41 (1.19)	−0.96 (1.12)	−1.42 (2.20)	6.751 (1;82) *	0.076	1.916 (1;82)	0.023	2.145 (1;82)	0.025	CG1 > AH-IMP (*d* = 1.46), MATH-IMP (*d* = 1.10)
TMOQ_t	0.10 (0.92)	−1.23 (1.11)	−0.45 (1.01)	−1.61 (0.71)	20.685 (1;117) ***	0.150	2.157 (1;117)	0.018	0.030 (1;117)	0.001	CG1 > AH/IMP (*d* = 1.34), MATH/IMP > AH/IMP+MATH/IMP (*d* = 1.19)

Note. All scores are standardized (z-scores). “CG1”, Control Group 1; “AH/IMP”, Attention/Hyperactivity Impairment group; “MATH/IMP”, Mathematical Impairment group; “TE”, Time Estimation task; “TR”, Time Reproduction task, “TD”, Time Discrimination task “TMOQ_c”, Time Management and Orienting Questionnaire child version; “TMOQ_p”, Time Management and Orienting Questionnaire parent version; “TMOQ_t”, Time Management and Orienting Questionnaire teacher version. “*d*”, Cohen’s d. *. *p* < 0.05; **. *p* < 0.01; ***. *p* < 0.001.

**Table 5:** Means, Standard Deviations, and ANCOVA Results for SoT Tests by Diagnostic Group (KBIT2-adjusted).

	CG2 (32.85% F)	ADHD (16.66%F)	DD (58.3%F)	ADHD +DD (66.66%F)	ADHD Effect		DD Effect		ADHD*DD		Contrasts
	M (SD)	M (SD)	M (SD)	M (SD)	F (dfs)	ɳ^2^p	F (dfs)	ɳ^2^p	F (dfs)	ɳ^2^p	CG2 vs. ADHD vs. DD vs. ADHD + DD
TE	−0.04 (1.06)	−0.37 (1.49)	−1.03 (2.77)	−1.96 (1.61)	1.470 (1;140)	0.010	5.817 (1;140) *	0.040	0.322 (1;140)	0.002	CG2 > DD (*d* = 0.51)
TR	0.11 (0.75)	−0.55 (1.19)	−0.13 (1.01)	−0.26 (0.98)	1.690 (1;135)	0.012	0.552 (1;135)	0.004	0.628 (1;135)	0.005	-
TD	0.10 (0.86)	−0.41 (1.25)	0.067 (0.95)	−0.81 (2.31)	4.218 (1;135) *	0.030	0.002 (1;135)	0.000	0.325 (1;135)	0.002	CG2 > ADHD (*d* = 0.49)
TMOQ_c	0.25 (0.93)	−0.33 (1.32)	−0.94 (1.75)	−0.70 (2.57)	0.148 (1; 115)	0.001	1.874 (1;115)	0.016	0.798 (1;115)	0.007	-
TMOQ_p	0.30 (1.01)	−1.32 (1.21)	0.29 (1.08)	−1.94 (1.57)	25.691 (1;93) ***	0.216	0.342 (1;93)	0.004	0.994 (1;93)	0.008	CG2 > ADHD (*d* = 1.43); DD > ADHD + DD (*d* = 1.95)
TMOQ_t	0.26 (0.78)	−0.82 (1.15)	−0.81 (1.20)	−0.87 (.)	1.006 (1;84)	0.012	0.771 (1;84)	0.009	0.898 (1;84)	0.011	-

Note. All scores are standardized (z-scores). “CG2”, Control Group 2; “ADHD”, Attention Deficit and Hyperactivity Disorder group; “DD”, Developmental Dyscalculia group; “TE”, Time Estimation task; “TR”, Time Reproduction task, “TD”, Time Discrimination task “TMOQ_c”, Time Management and Orienting Questionnaire child version; “TMOQ_p”, Time Management and Orienting Questionnaire parent version; “TMOQ_t”, Time Management and Orienting Questionnaire teacher version. “*d*”, Cohen’s d. *. *p* < 0.05; ***. *p* < 0.001.

## Data Availability

The data are available on the Open Science Framework (OSF) at the following link: https://osf.io/nhe52/overview?view_only=7a44e7de56b149f1812d47d7879716a6. Accessed on 22 January 2026.

## References

[1] Grondin S. (2010). Timing and time perception: A review of recent behavioral and neuroscience findings and theoretical directions. Atten. Percept. Psychophys..

[2] Droit-Volet S. (2016). Development of time. Curr. Opin. Behav. Sci..

[3] Helfrich H.E. (2003). Time and Mind II: Information Processing Perspectives.

[4] Forman H. (2015). Events and children’s sense of time: A perspective on the origins of everyday time-keeping. Front. Psychol..

[5] Buzi G., Eustache F., Droit-Volet S., Desaunay P., Hinault T. (2024). Towards a neurodevelopmental cognitive perspective of temporal processing. Commun. Biol..

[6] Coull J., Nobre A. (2008). Dissociating explicit timing from temporal expectation with fMRI. Curr. Opin. Neurobiol..

[7] Allman M.J., Pelphrey K.A., Meck W.H. (2012). Developmental neuroscience of time and number: Implications for autism and other neurodevelopmental disabilities. Front. Integr. Neurosci..

[8] Block R.A., Zakay D., Hancock P.A. (1999). Developmental changes in human duration judgments: A meta-analytic review. Dev. Rev..

[9] Droit-Volet S. (2011). Child and time. Multidisciplinary Aspects of Time and Time Perception: COST TD0904 International Workshop, Athens, Greece, 7–8 October 2010.

[10] Zélanti P.S., Droit-Volet S. (2011). Cognitive abilities explaining age-related changes in time perception of short and long durations. J. Exp. Child Psychol..

[11] Droit-Volet S., Zélanti P. (2013). Development of time sensitivity: Duration ratios in time bisection. Q. J. Exp. Psychol..

[12] Tombini G., Tobia V., Ghislanzoni L., Gambarini A., Ogliari A., Marzocchi G.M. (2024). Teachers’ report of sense of time in kindergarten predicts children’s time-processing skills in first grade. Int. J. Psychol..

[13] Barkley R.A. (1997). Behavioral inhibition, sustained attention, and executive functions: Constructing a unifying theory of ADHD. Psychol. Bull..

[14] Janowsky J.S., Shimamura A.P., Squire L.R. (1989). Memory and metamemory: Comparisons between patients with frontal lobe lesions and amnesic patients. Psychobiology.

[15] Kerns K.A., McInerney R.J., Wilde N.J. (2001). Time reproduction, working memory, and behavioral inhibition in children with ADHD. Child Neuropsychol..

[16] Lewis P.A., Miall R.C. (2006). Remembering the time: A continuous clock. Trends Cogn. Sci..

[17] Rammsayer T.H. (1999). Neuropharmacological evidence for different timing mechanisms in humans. Q. J. Exp. Psychol. Sect. B.

[18] Diamond A. (2013). Executive functions. Annu. Rev. Psychol..

[19] Block R.A., Hancock P.A., Zakay D. (2010). How cognitive load affects duration judgments: A meta-analytic review. Acta Psychol..

[20] Buhusi C.V., Meck W.H. (2009). Relative time sharing: New findings and an extension of the resource allocation model of temporal processing. Philos. Trans. R. Soc. B Biol. Sci..

[21] De Lurdes Delgado M., Droit-Volet S. (2007). Testing the representation of time in reference memory in the bisection and the generalization task: The utility of a developmental approach. Q. J. Exp. Psychol..

[22] Gibbon J., Church R.M., Meck W.H. (1984). Scalar timing in memory. Ann. N. Y. Acad. Sci..

[23] Gibbon J. (1977). Scalar expectancy theory and Weber’s law in animal timing. Psychol. Rev..

[24] Nombre C., Coull J.T. (2011). Attention and Time.

[25] Rubia K., Smith A. (2004). The neural correlates of cognitive time management: A review. Acta Neurobiol. Exp..

[26] Thomas E.A., Weaver W.B. (1975). Cognitive processing and time perception. Percept. Psychophys..

[27] Treisman M. (1963). Temporal discrimination and the indifference interval: Implications for a model of the “internal clock”. Psychol. Monogr. Gen. Appl..

[28] Glicksohn J., Myslobodsky M.S. (2006). Timing the Future: The Case for A Time-Based Prospective Memory.

[29] Labelle M.A., Graf P., Grondin S., Gagné-Roy L. (2009). Time-related processes in time-based prospective memory and in time-interval production. Eur. J. Cogn. Psychol..

[30] Roy M.M., Christenfeld N.J. (2008). Effect of task length on remembered and predicted duration. Psychon. Bull. Rev..

[31] Thomas K.E., Handley S.J., Newstead S.E. (2007). The role of prior task experience in temporal misestimation. Q. J. Exp. Psychol..

[32] Carelli M.G., Forman H., Mäntylä T. (2008). Sense of time and executive functioning in children and adults. Child Neuropsychol..

[33] Mäntylä T., Carelli M.G., Forman H. (2007). Time monitoring and executive functioning in children and adults. J. Exp. Child Psychol..

[34] Cappelletti M., Freeman E.D., Butterworth B.L. (2011). Time processing in dyscalculia. Front. Psychol..

[35] Smith A., Taylor E., Warner Rogers J., Newman S., Rubia K. (2002). Evidence for a pure time perception deficit in children with ADHD. J. Child Psychol. Psychiatry.

[36] Chan A.S., Sze S.L., Cheung M.C. (2023). Temporal processing tele-intervention improves language, attention, and memory in children with neurodevelopmental disorders. Digit. Health.

[37] Moll K., Göbel S.M., Gooch D., Landerl K., Snowling M.J. (2016). Cognitive risk factors for specific learning disorder: Processing speed, temporal processing, and working memory. J. Learn. Disabil..

[38] Mullin A.P., Gokhale A., Moreno-De-Luca A., Sanyal S., Waddington J.L., Faundez V. (2013). Neurodevelopmental disorders: Mechanisms and boundary definitions from genomes, interactomes and proteomes. Transl. Psychiatry.

[39] APA (2022). Diagnostic and Statistical Manual of Mental Disorders.

[40] Astle D.E., Holmes J., Kievit R., Gathercole S.E. (2022). Annual Research Review: The transdiagnostic revolution in neurodevelopmental disorders. J. Child Psychol. Psychiatry.

[41] Insel T.R., Cuthbert B.N., Garvey M.A., Heinssen R., Pine D.S., Quinn K., Sanislow C., Wang P. (2010). Research domain criteria (RDoC): Toward a new classification framework for research on mental disorders. Am. J. Psychiatry.

[42] Ptacek R., Weissenberger S., Braaten E., Klicperova-Baker M., Goetz M., Raboch J., Vnukova M., Stefano G.B. (2019). Clinical implications of the perception of time in attention deficit hyperactivity disorder (ADHD): A review. Med. Sci. Monit. Int. Med. J. Exp. Clin. Res..

[43] Barkley R.A., Grodzinsky G., DuPaul G.J. (1992). Frontal lobe functions in attention deficit disorder with and without hyperactivity: A review and research report. J. Abnorm. Child Psychol..

[44] Cubillo A., Halari R., Smith A., Taylor E., Rubia K. (2012). A review of fronto-striatal and fronto-cortical brain abnormalities in children and adults with Attention Deficit Hyperactivity Disorder (ADHD) and new evidence for dysfunction in adults with ADHD during motivation and attention. Cortex.

[45] Noreika V., Falter C.M., Rubia K. (2013). Timing deficits in attention-deficit/hyperactivity disorder (ADHD): Evidence from neurocognitive and neuroimaging studies. Neuropsychologia.

[46] Rubia K., Halari R., Mohammad A.M., Taylor E., Brammer M. (2011). Methylphenidate normalizes frontocingulate underactivation during error processing in attention-deficit/hyperactivity disorder. Biol. Psychiatry.

[47] Hart H., Marquand A.F., Smith A., Cubillo A., Simmons A., Brammer M., Rubia K. (2014). Predictive neurofunctional markers of attention-deficit/hyperactivity disorder based on pattern classification of temporal processing. J. Am. Acad. Child Adolesc. Psychiatry.

[48] Nakao T., Radua J., Rubia K., Mataix-Cols D. (2011). Gray matter volume abnormalities in ADHD: Voxel-based meta-analysis exploring the effects of age and stimulant medication. Am. J. Psychiatry.

[49] Rubia K., Halari R., Cubillo A., Mohammad A.M., Brammer M., Taylor E. (2009). Methylphenidate normalises activation and functional connectivity deficits in attention and motivation networks in medication-naive children with ADHD during a rewarded continuous performance task. Neuropharmacology.

[50] Toplak M.E., Dockstader C., Tannock R. (2006). Temporal information processing in ADHD: Findings to date and new methods. J. Neurosci. Methods.

[51] Sonuga-Barke E.J. (2002). Psychological heterogeneity in AD/HD—A dual pathway model of behaviour and cognition. Behav. Brain Res..

[52] Sonuga-Barke E.J., Taylor E., Sembi S., Smith J. (1992). Hyperactivity and delay aversion—I. The effect of delay on choice. J. Child Psychol. Psychiatry.

[53] Sonuga-Barke E.J., Sergeant J.A., Nigg J., Willcutt E. (2008). Executive dysfunction and delay aversion in attention deficit hyperactivity disorder: Nosologic and diagnostic implications. Child Adolesc. Psychiatr. Clin. N. Am..

[54] Schachar R., Tannock R., Marriott M., Logan G. (1995). Deficient inhibitory control in attention deficit hyperactivity disorder. J. Abnorm. Child Psychol..

[55] McGee R., Brodeur D., Symons D., Andrade B., Fahie C. (2004). Time perception: Does it distinguish ADHD and RD children in a clinical sample?. J. Abnorm. Child Psychol..

[56] Sonuga-Barke E., Bitsakou P., Thompson M. (2010). Beyond the dual pathway model: Evidence for the dissociation of timing, inhibitory, and delay-related impairments in attention-deficit/hyperactivity disorder. J. Am. Acad. Child Adolesc. Psychiatry.

[57] Mioni G., Capodieci A., Biffi V., Porcelli F., Cornoldi C. (2019). Difficulties of children with symptoms of attention-deficit/hyperactivity disorder in processing temporal information concerning everyday life events. J. Exp. Child Psychol..

[58] Zheng Q., Cheng Y.Y., Sonuga-Barke E., Shum K.K.M. (2022). Do executive dysfunction, delay aversion, and time perception deficit predict ADHD symptoms and early academic performance in preschoolers. Res. Child Adolesc. Psychopathol..

[59] Toffalini E., Giofrè D., Cornoldi C. (2017). Strengths and weaknesses in the intellectual profile of different subtypes of specific learning disorder: A study on 1049 diagnosed children. Clin. Psychol. Sci..

[60] Casini L., Pech-Georgel C., Ziegler J.C. (2018). It’s about time: Revisiting temporal processing deficits in dyslexia. Dev. Sci..

[61] Walsh V. (2003). A theory of magnitude: Common cortical metrics of time, space and quantity. Trends Cogn. Sci..

[62] Piazza M., Izard V. (2009). How humans count: Numerosity and the parietal cortex. Neuroscientist.

[63] Tobia V., Rinaldi L., Marzocchi G.M. (2018). Time processing impairments in preschoolers at risk of developing difficulties in mathematics. Dev. Sci..

[64] Träff U., Olsson L., Östergren R., Skagerlund K. (2017). Heterogeneity of developmental dyscalculia: Cases with different deficit profiles. Front. Psychol..

[65] (2010). Legge 8 Ottobre 2010, n. 170 (Nuove Norme in Materia di Disturbi Specifici di Apprendimento in Ambito Scolastico). Gazzetta Ufficiale della Repubblica Italiana, Serie Generale, n. 244 (18 Ottobre 2010). https://www.gazzettaufficiale.it/eli/id/2010/10/18/010G0192/sg.

[66] Dewey D. (2018). What is comorbidity and why does it matter in neurodevelopmental disorders?. Curr. Dev. Disord. Rep..

[67] Cortesi F., Polenghi I., Gambarini A., Marzocchi G.M., Mioni G., Ogliari A., Toffalini E., Tobia V. (2026). Time processing skills in individuals with math impairment and Developmental Dyscalculia. Res. Dev. Disabil..

[68] Stojić S., Topić V., Nadasdy Z. (2023). Children and adults rely on different heuristics for estimation of durations. Sci. Rep..

[69] Peirce J., Gray J.R., Simpson S., MacAskill M., Höchenberger R., Sogo H., Kastman E., Lindeløv J.K. (2019). PsychoPy2: Experiments in behavior made easy. Behav. Res. Methods.

[70] Rosselló B., Servera M. (2015). Analysis of the Time Management Scale for Teachers and their application in ADHD. J. Clin. Psychol. Child. Adolesc..

[71] Tobia V., Bonifacci P., Bernabini L., Marzocchi G.M. (2019). Teachers, not parents, are able to predict time processing skills in preschoolers. Br. J. Dev. Psychol..

[72] Marzocchi G.M., Re A.M., Cornoldi C. (2021). BIA-R—Batteria Italiana per l’ADHD—Revised. Valutazione dei Bambini con Deficit di Attenzione-Iperattività.

[73] Kaufman A.S., Kaufman N.L. (2004). KBIT-2: Kaufman Brief Intelligence Test.

[74] Cornoldi C., Mammarella I.C., Caviola S. (2020). ACMT 3 6-14: Prove per la Clinica.

[75] Biancardi A., Nicoletti C., Bachmann C. (2016). BDE 2-Batteria Discalculia Evolutiva: Test per la Diagnosi dei Disturbi Dell’Elaborazione Numerica e del Calcolo in Età Evolutiva–8-13 Anni.

[76] Bauermeister J.J., Barkley R.A., Martínez J.V., Cumba E., Ramírez R.R., Reina G., Matos M., Salas C.C. (2005). Time estimation and performance on reproduction tasks in subtypes of children with attention deficit hyperactivity disorder. J. Clin. Child Adolesc. Psychol..

[77] Valko L., Schneider G., Doehnert M., Müller B., Landgraf M., Rothenberger A. (2010). Time processing in children and adults with ADHD. J. Neural Transm..

[78] Yehoshua T., Shalev L., Mevorach C. (2005). The diversity of attention deficits in ADHD: The prevalence of four cognitive factors in ADHD versus controls. J. Learn. Disabil..

[79] Mette C. (2023). Time perception in adult ADHD: Findings from a decade—A review. Int. J. Environ. Res. Public Health.

[80] Butterworth B. (2005). The development of arithmetical abilities. J. Child Psychol. Psychiatry.

[81] Dormal V., Seron X., Pesenti M. (2006). Numerosity–duration interference: A Stroop experiment. Acta Psychol..

[82] Kaufmann L. (2008). Dyscalculia: Neuroscience and education. Educ. Res..

[83] Kotov R., Krueger R.F., Watson D., Cicero D.C., Conway C.C., DeYoung C.G., Eaton N.R., Forbes M.K., Hallquist M.N., Latzman R.D. (2021). The Hierarchical Taxonomy of Psychopathology (HiTOP): A Quantitative Nosology Based on Consensus of Evidence. Annu. Rev. Clin. Psychol..

[84] Michelini G., Carlisi C.O., Eaton N.R., Elison J.T., Haltigan J.D., Kotov R., Krueger R.F., Latzman R.D., Li J.J., Levin-Aspenson H.F. (2024). Where do neurodevelopmental conditions fit in transdiagnostic psychiatric frameworks? Incorporating a new neurodevelopmental spectrum. World Psychiatry.

[85] Lense M.D., Ladányi E., Rabinowitch T.C., Trainor L., Gordon R. (2021). Rhythm and timing as vulnerabilities in neurodevelopmental disorders. Philos. Trans. R. Soc. B Biol. Sci..

[86] Skagerlund K., Träff U. (2014). Development of magnitude processing in children with developmental dyscalculia: Space, time, and number. Front. Psychol..

[87] Cuthbert B.N., Insel T.R. (2013). Toward the future of psychiatric diagnosis: The seven pillars of RDoC. BMC Med..

[88] Poletti M., Preti A., Raballo A. (2025). Editorial Perspective: Issues for DSM 6—An Alternative Model for Neurodevelopmental Disorders to enhance nosological validity and clinical utility. J. Child Psychol. Psychiatry.

[89] Sonuga-Barke E.J.S. (2022). ‘If it don’t fit, don’t force it?’ if real-world, complex clinical decisions are intrinsically categorical can dimensional systems add value? Reflections on Lahey, Tiemeier & Krueger (2022). JCPP Adv..

[90] Oliveira J., Rocha T., Barroso J. (2025). A Personalized Digital Solution to Assist Task Organization and Time Management for People with Attention Deficit/Hyperactivity Disorder (ADHD). International Conference of the Association for the Advancement of Assistive Technology in Europe.

[91] Lakens D. (2022). Sample size justification. Collabra Psychol..

